# Retro-Inverso Carbohydrate Mimetic Peptides with Annexin1-Binding Selectivity, Are Stable *In Vivo*, and Target Tumor Vasculature

**DOI:** 10.1371/journal.pone.0080390

**Published:** 2013-12-02

**Authors:** Xinyi Chen, Zhuoyang Fan, Yanzuo Chen, Xiaoling Fang, Xianyi Sha

**Affiliations:** 1 Key Laboratory of Smart Drug Delivery (Fudan University), Ministry of Education, Department of Pharmaceutics, School of Pharmacy, Fudan University, Shanghai, China; National Cancer Institute at Frederick, United States of America

## Abstract

Previous research suggests that carbohydrate mimetic peptide IF7 (IFLLWQR) has an excellent targeting property to annexin1 (Anxa1), a specific marker on the tumor endothelium. However, IF7 is susceptible to proteolysis and has a poor stability *in vivo*. We prepared a D-amino acid, reverse sequence peptide of IF7, designated RIF7, to confer protease resistance while retaining bioactivity. Experimental results indicate that RIF7 had significantly increased stability and an increased receptor binding affinity than IF7, and this new moiety may represent a clinically relevant vehicle for anticancer drugs.

## Introduction

Targeted therapeutics hold great promise for cancer therapy compared to conventional chemotherapy, providing improved efficacy and fewer adverse effects [1]. Some success has been achieved in the application of targeted ligands, including antibodies, single-chain Fv fragments [2], [3], peptides [4], [5], small molecules [6], and aptamers [7], [8]. Among these example, peptides-or peptide mimetics-are emerging as the most often used targeted ligands. They have multiple favorable characteristics, including a lack of immunogenicity as well as easy low cost synthesis.

Carbohydrate structures on the tumor cell surface are closely associated with cancer malignancy and these structures suppress or promote tumor growth by interacting with specific highly expressed proteins on tumor cells [9]. Such information has driven investigations of targeted tumor therapy which can hypothetically be achieved by exploiting such carbohydrate structures as targets; however, carbohydrate-based drug discovery has not been fully explored due to an inability to synthesize complex carbohydrate structures [10]. Consequently, peptides as structural or functional mimetics of carbohydrates with the same binding sites (carbohydrate mimetic peptides) are of intense interest [11]. However, the high susceptibility of peptides to proteolysis continues to be an obstacle to the development of carbohydrate mimetic peptides [12], [13].

Hatakeyama's laboratory used peptide-displaying phage technology to generate a carbohydrate mimetic peptide with the consensus sequence IFLLWQR, which was designated IF7 [14]. IF7 has a special affinity for the tumor vasculature and functions as an efficient anticancer drug delivery vehicle, slowing tumor growth. Experiments confirmed that IF7 specifically binds to a 15-kDa fragment of annexin1 (Anxa1). Anxa1 is a member of the large annexin family of cytosolic proteins with calcium-dependent associations with cell membranes. Previous research indicated that Anxa1 was upregulated on the tumor endothelium [15]. However, like all L-amino acid peptides, IF7 was susceptible to proteolysis and had poor stability *in vivo* despite excellent tumor-targeting activity. IF7 was detected in the tumor within 1 min of injection in mice, reached a plateau in 9 min, but remained high only for 40 min. To increase the efficacy of IF7, a popular pharmacological technique of replacing L- amino acids by D-amino acids while reversing the primary sequence of the peptide was attempted [16]. Data indicate that several retro-inverso peptides had increased stability and improved bioactivity compared with their native structures, and this has been confirmed for a number of peptides, including enkephalin, glutathione, Substance P, gastrin, and atrial natiuretic peptide [16], [17].

Thus, we employed D-configuration technology and synthetized retro-inverso peptides of IF7 to enhance stability. Retro-inverso—or retro-all-D retroenantio—peptide analogues not only have peptide bonds that are directionally reversed compared to their parent peptide, but also, the L-amino acids have been replaced with D-amino acids. Such peptides obtained from this peptide-bond reversal and chiral inversion are promising peptide-based therapeutics with greater resistance to peptidases. Nevertheless, remaining questions about whether the D-configured peptides retain biological activity are presently unanswered.

**Figure 1:**
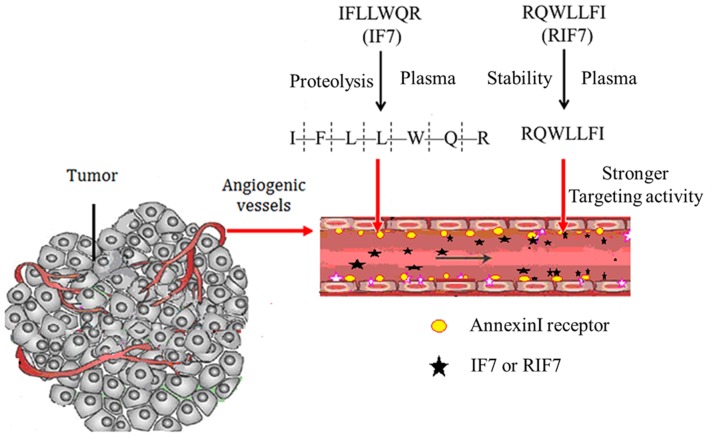
Design and different features of IF7 and RIF7 in the tumor vasculature.

Therefore, to address this uncertainty, we synthetized retro-inverso IF7, or RIF7 (RQWLLFI) and tested our hypothesis that this compound would not be susceptible to proteolysis, retaining specificity to Anxa1 compared to IF7, which is easily hydrolyzed (Fig. 1). TMR (5-carboxytetramethylrhodamine) was utilized as a fluorescent probe to evaluate uptake of RIF7 by A549 epithelial cells, which highly express Anxa1 after induction with phorbol myristate acetate (PMA) treatment [18]. We also measured the *in vivo* tumor targeting activity of RIF7 after intravenous injection. Finally, inhibition assays were used to study Anxa1 targeting of RIF7. We report that RIF7 is stable *in vivo* and retains bioactivity, and we propose that RIF7 is a promising tumor targeting moiety for tumor therapy.

## Materials and Methods

### Peptides and antibodies

Fmoc-D-amino acids {Phe, Leu, Trp(boc), Gln(trt) and Arg(pbf)}, 1-Hydroxybenzotrizole (HOBT), Fmoc-D-Ile-Wang Resin and 5-carboxytetramethylrhodamine (TMR) were purchased from GL Biochem (Shanghai) Ltd., China. All other chemicals used in synthesis were of reagent grade, were obtained from Sigma-Aldrich (St. Louis, MO, USA) and were used without further purification.

RIF7 and IF7 peptides were obtained by solid-phase peptide synthesis using D- or L-amino acids. Installation of a fluorescent probe molecule, TMR was accomplished using solid phase synthesis. Fmoc-D-Ile-Wang Resin was added to the reaction column and pretreated with N, N-di-isopropylethylamine (DIEA). For capping and deprotection procedures, a solution of Fmoc-D-Phe-OH, HOBt, and DIC in DMF was prepared. This solution was then added to the reaction column and swabbed off after the reaction was complete. Then, the column was washed with DMF. The other five Fmoc-amino acids were conjugated with the same procedures.

Prior to cleavage, the resin was washed with DCM and dried under a vacuum. The desired peptide was cleaved from the resin after being shaken under N_2_ with reagent K (TFA: 82.5, water: 5, phenol: 5, thioanisole: 5, and EDT: 2.5) for 3 h. The crude product in the cleavage mixture was precipitated with cold ether, collected by centrifugation, washed three times with cold ether, and then finally purified by HPLC.

The TMR-labeled peptides were cleaved from the resin and isolated. To determine the purity of the synthesis, a sample of the solution was injected onto a Shimadzu LC-15C HPLC equipped with a Vydac C18, 218TP54 column (4.6×250 mm, Welch Materials, Inc. China), which was eluted by a 25 min (1 ml/min) linear gradient of 40–65% aqueous acetonitrile containing 0.1% trifluoroacetic acid. Eluted peptides were detected (absorbance = 220 nm) with a UV monitor.

For the inhibition studies, rabbit anti-Anxa1 antibody and normal rabbit IgG were purchased from Santa Cruz Biotechnology, Inc. (Santa Cruz, CA).

### Cell lines and culture conditions

Murine melanoma cells (B16-F10) and the MDR human oral carcinoma KBv cell line were obtained from the Chinese Academy of Sciences Cells Bank, Shanghai, China. They were cultured in RPMI Medium 1640 supplemented with 10% fetal bovine serum (FBS), 100 U/mL penicillin and 0.1 mg/mL streptomycin solution at 37°C under 5% CO_2_.

The human adenocarcinoma cell line A 549 (obtained from the Chinese Academy of Sciences Cells Bank, Shanghai, China) was expanded and maintained in special Dulbecco's modified Eagle medium (DMEM) supplemented with 10% heat-inactivated FBS, 100 U/mL penicillin, 0.1 mg/mL streptomycin and cultured at 37°C under a humidified atmosphere containing 5% CO_2_. RPMI Medium 1640,FBS, DMEM, Trypsin-EDTA (0.25%), and penicillin-streptomycin were purchased from Gibco BRL (Gaithersberg, MD). All experiments were performed on cells that were in a logarithmic growth phase.

### Animals

BALB/C mice (male, 5 weeks-of-age, 18–22 g) and BALB/c nude mice (male, 5 weeks-of-age, 18–22 g) were supplied by the Department of Experimental Animals, Fudan University (Shanghai, China), and maintained under standard housing conditions with water and food available. All animal experiments were carried out in strict accordance with guidelines evaluated and approved by the ethics committee of Fudan University (Shanghai, China). All animal surgery was performed under sodium pentobarbital anesthesia, and all efforts were made to minimize suffering.

### Stability of TMR labeled IF7 (TMR-IF7) and RIF7 (TMR-RIF7) in mouse plasma

Mouse blood was collected in serum-separation tubes containing heparin sodium and immediately centrifuged at 10,000 rpm for 10 min. Fresh mouse plasma was then removed and stored for further analysis. Then, 200 µg of TMR-RIF7 and TMR-IF7 was dissolved in 10 µl DMSO, added to 1 ml of the fresh plasma, and incubated at 37°C. A 50 µl aliquot was withdrawn from the incubation mixture at the time indicated, added to 450 µl acetonitrile: water (50∶50) containing 0.1% TFA, centrifuged at 5,000 rpm for 10 min to remove Insoluble material, and then the supernatant was analyzed by HPLC as described previously in §2.2. Elution was monitored (excitation λ = 543 nm; emission λ = 570 nm). The relative amount of TMR-RIF7 and TMR-IF7 that remained in the mouse plasma was incubated at 37°C.

### Cell-binding study

A549 cells were seeded (5×10^3^ cells/well) in 96-well plates (Corning Coaster, Tokyo, Japan) and incubated for 12 h. Cells were incubated with serum free medium (special Dulbecco's modified Eagle medium supplemented with 100 U/mL penicillin, 100 mg/mL streptomycin) containing TMR-RIF7, TMR-IF7, or TMR-RQ7 (TMR conjugated to reverse IF7: sequence RQWLLFI, composed of L-amino acids) at the concentration of 10 µg/ml. In addition, to verify the stability of modified peptides, TMR-IF7 and TMR-RIF7 were incubated with complete medium (special Dulbecco's modified Eagle medium supplemented with 10% heat-inactivated FBS, 100 U/mL penicillin, 100 mg/mL streptomycin) for 10 min at 37°C in advance of serum free medium. The solution was removed and cells were washed three times with ice-cold PBS (pH 7.4) and then visualized under a fluorescent microscope (Leica DMI 4000B, Germany).

For inhibition assays, anti-Anxa1 antibody or control normal rabbit IgG (4 µg each) was added to each well and incubated for 30 min. TMR-IF7 or TMR-RIF7 (10 µg/ml) were then added and incubated at 37°C for 30 min. The solution was removed and cells were washed three times with ice-cold PBS (pH 7.4) and then visualized under a fluorescent microscope (Leica DMI 4000B, Germany).

For direct competition studies, non-labeled IF7 or RIF7 (10 µg/ml) was added to each well and incubated for 30 min. TMR-IF7 or TMR-RIF7 (10 µg/ml) were then added and incubated at 37°C for 30 min. The solution was removed and the cells were washed three times with ice-cold PBS (pH 7.4) and then visualized under a fluorescent microscope (Leica DMI 4000B, Germany). The fluorescent signal intensity of each well was quantified using an F-7000 FL spectrophotometer.

### Pulmonary and subcutaneous tumor implantation

B16-F10 cells (7.5×10^5^ cells suspended in 250 µl PBS) were injected intravenously into BALB/c mice to establish a pulmonary metastatic melanoma model. Specifically, KBv cells (5×10^6^cells suspended in 200 ml cell culture medium) were inoculated subcutaneously into the right hind legs of nude mice to produce a subcutaneous KBv xenograft model.

### In vivo tumor-targeting activity by TMR-RIF7

*Ex vivo* real-time fluorescence imaging analysis was used to evaluate the tumor targeting activity of TMR-RIF7. One week after inoculation, TMR-IF7 or TMR-RIF7 (200 µl, 0.5 mg/ml in 5% glucose solution) was injected through the tail vein of mice bearing pulmonary tumors. The mice were then sacrificed and perfused intracardially with normal saline to compare tissue and tumor distribution of TMR-RIF7. Tumor-bearing lungs and other major organs, including hearts, livers, spleens and kidneys were removed, washed with saline, and imaged using a Cambridge Research & Instrumentation (CRi) *in vivo* imaging system (CRi, MA, USA). When subcutaneous KBv tumors were grown to a palpable size (about 8–10 mm diameter), the same process was carried out to evaluate tissue and tumor distribution of TMR-RIF7 in subcutaneous tumor-bearing nude mice. Next, 20 µg of each rabbit anti-Anxa1 antibody (N-19) or rabbit IgG antibody was injected 15 min prior to TMR-IF7 or TMR-RIF7 injection for inhibition assays in pulmonary tumor-bearing mice. Then, resulting images (spectral cube, containing a spectrum at every pixel) were loaded into Maestro™ 2.10 software and analyzed.

### Statistical analysis

Data are presented as the mean±standard deviation. Significance among groups were determined by one-way analysis of variance (ANOVA), followed by Bonferroni *post hoc* analysis for comparisons between individual groups. A value of p<0.05 was considered to be significant.

## Results and Discussion

### Characterization of TMR-IF7 and TMR-RIF7

**Figure 2:**
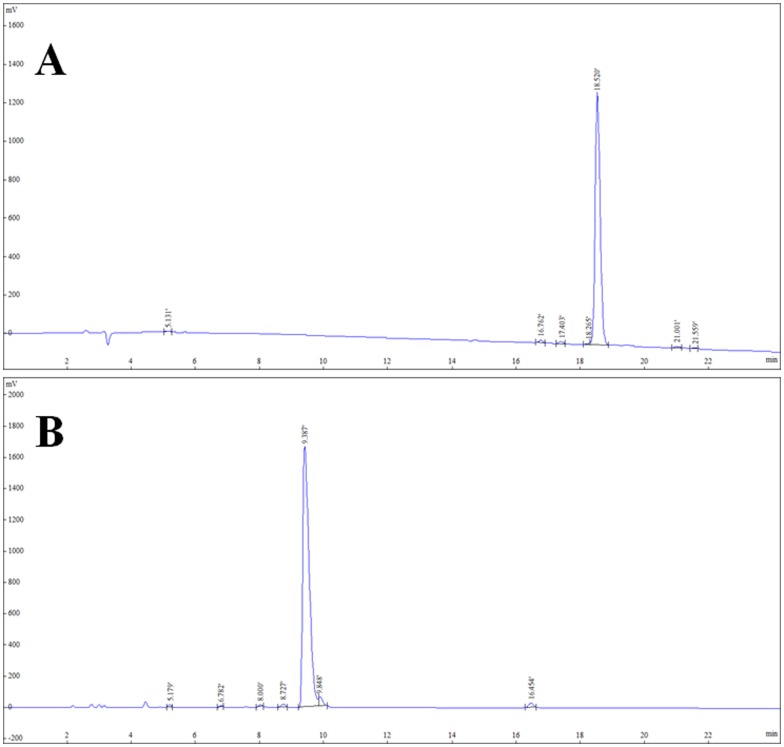
Spectrum of reversed phase-HPLC analysis of TMR-IF7 (A) and TMR-RIF7 (B).

IF7 (IFLLWQR) and RIF7 (RQWLLFI) peptides were chemically synthesized and conjugated with fluorescent TMR and the successful conjugation TMR-IF7 and TMR-RIF7 was confirmed by mass spectroscopic analysis. The purity of each was confirmed using reversed phase-HPLC (Fig. 2 A, B), and both were confirmed to have purity greater than 95%.

IF7 and RIF7 have different peak times although they are composed of identical amino acids, suggesting distinct physicochemical properties. RPHPLC profiles of the two isomeric peptides indicate that the RIF7 peptide (RQWLLFI) eluted earlier than the IF7 peptide (IFLLWQR), likely due to the binding of four trifluoroacetate (TFA) anions to arginine at the N-terminus in the RIF7 peptide. In contrast, three TFA anions bind to arginine at the C-terminus in the IF7 peptide.

### Reversed phase-HPLC analysis of TMR-IF7 (A) and TMR-RIF7 (B)

**Figure 3:**
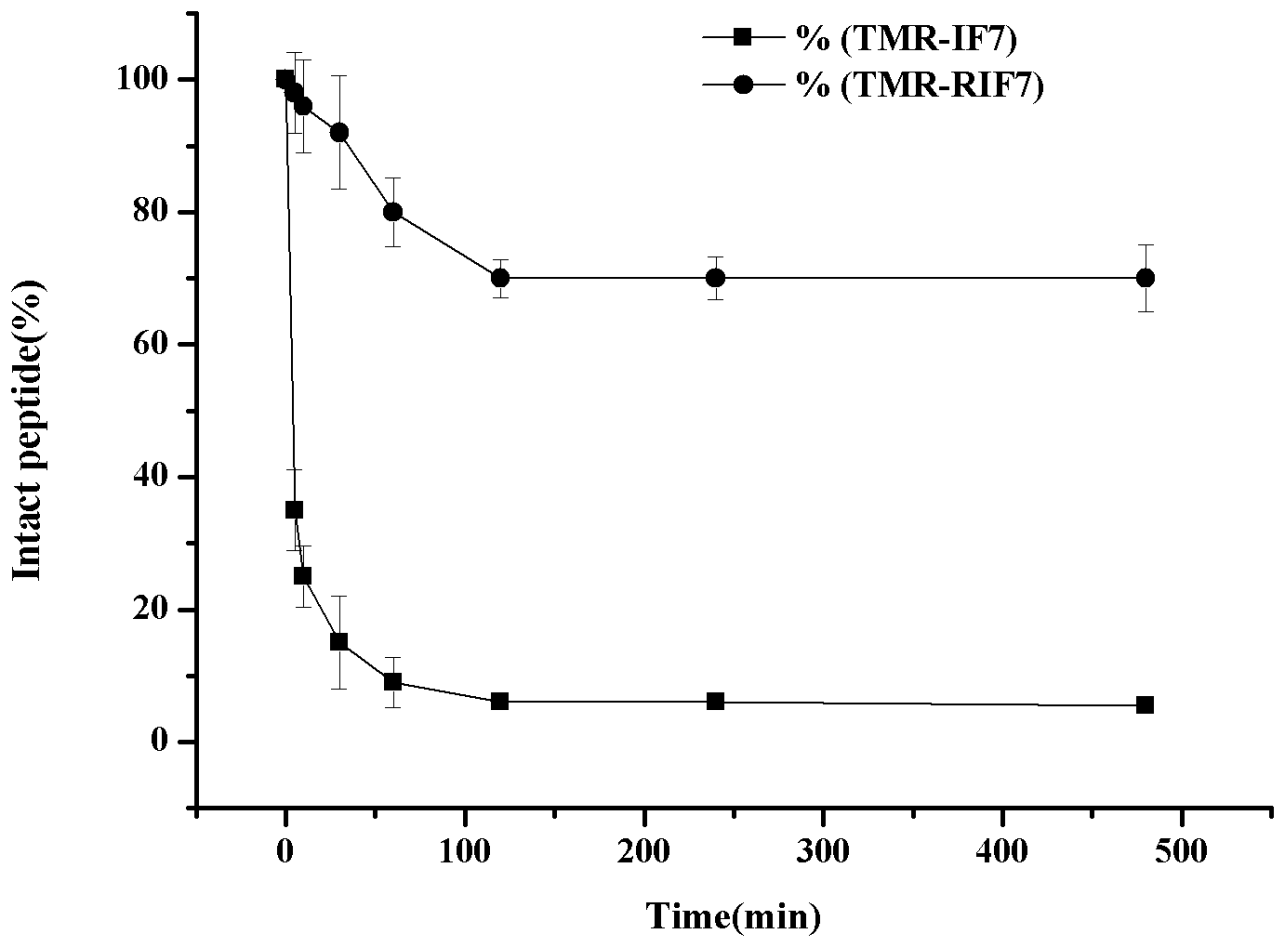
Remaining intact TMR-IF7(•) and TMR-RIF7(▪) in mouse plasma 37±0.5°C. Each point represents the mean ± SD of at least three experiments.

HPLC was used to evaluate the susceptibility of TMR-RIF7 and TMR-IF7 to mouse plasma proteases. As described in Fig. 3, TMR-IF7 rapidly degraded when incubated with mouse plasma, whereas TMR-RIF7 did not change significantly when incubated in the same manner. Specifically, ∼70% of the modified peptide remained intact within 6 h of plasma incubation and more than 80% of the unmodified peptide was degraded within 30 min. These observations suggest that retro-inverso modification can protect peptides against proteolysis and substantially improve peptide stability.

Because rapid protease degradation is a major obstacle for therapeutic use of peptides [13], enhanced stability is of great interest. Protease protection can be conferred by substituting a surrogate unit for the peptide bond [19] or replacing L-amino acids residues with D-amino acids or with unnatural residues (e.g., sarcosine and β-alanine). Most naturally occurring proteases cannot cleave D-amino acid residues and nonpeptide bonds [17], [20]. If all or some of the peptide bonds are reversed (NH-CO instead of CO-NH) in the modified segment, then retro-inverso peptides were confirmed to be products [21], [22].

### Cell binding of TMR-labeled peptides

**Figure 4:**
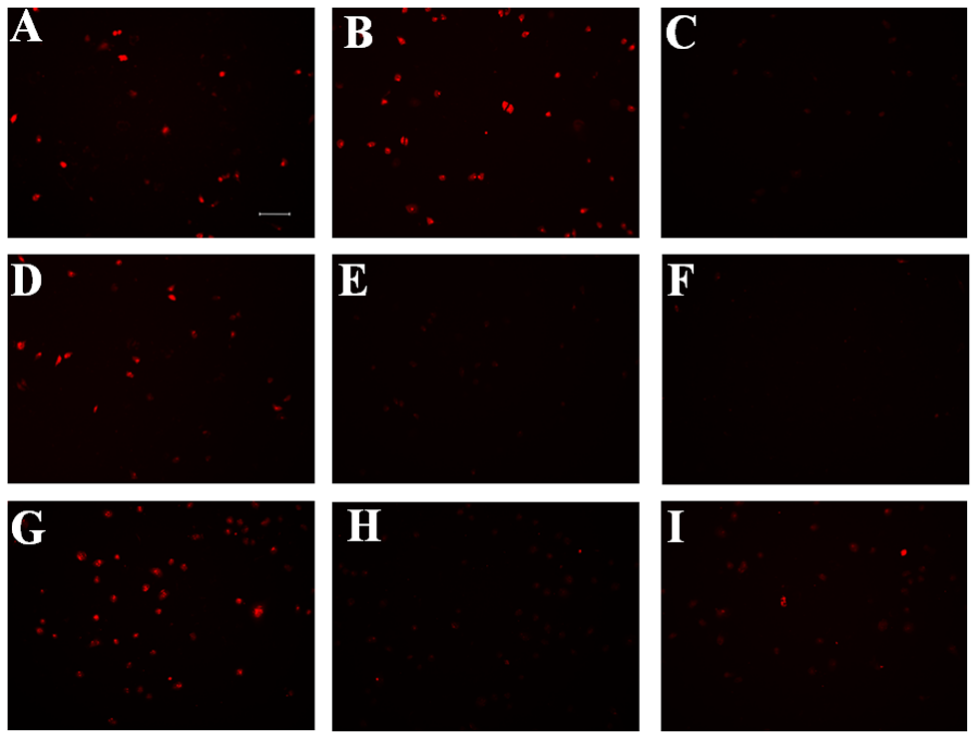
Qualitative analysis of peptide binding to A549 cells after 30 min of incubation. Fluorescent images for TMR-IF7 (A), TMR-RIF7 (B), TMR-RQ7 (C), TMR-IF7 plus normal rabbit IgG (D), TMR-RIF7 plus normal rabbit IgG (G), TMR-IF7 plus anti-Anxa1 antibody (E), TMR-RIF7 plus anti-Anxa1 antibody (H). TMR-IF7 (F) and TMR-RIF7 (I) were incubated with complete medium for 10 min prior to incubation with cells. Original magnification: ×20. Bar represents 100 µm.

**Figure 5:**
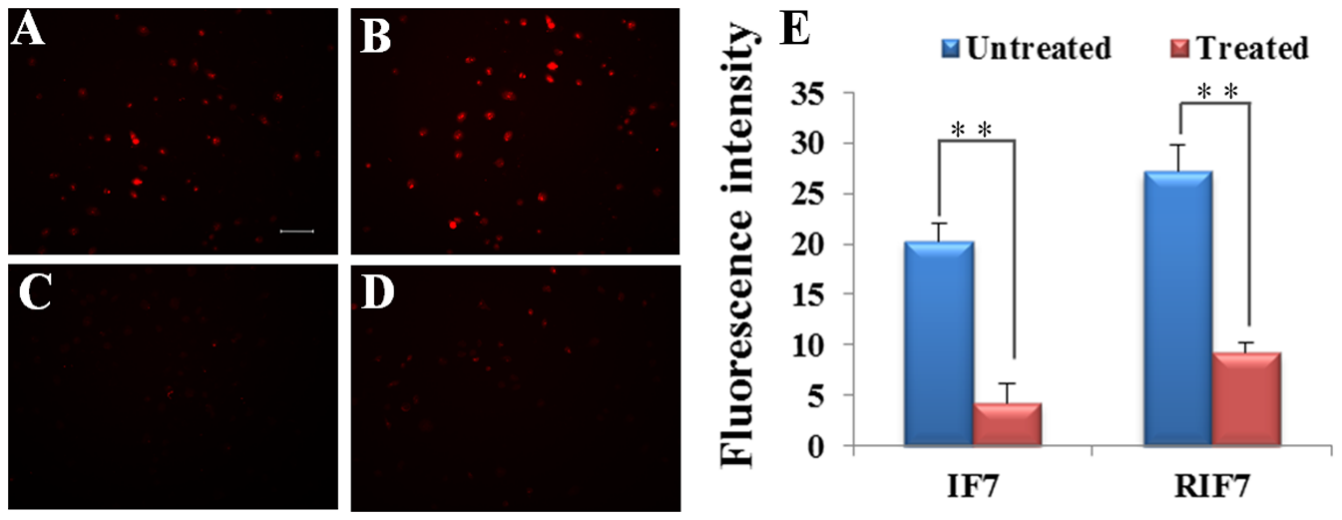
Quantitative cell-binding was measured using an F-7000 FL spectrophotometer. Data are presented as means ±SD (n = 3). **: *P*<0.01, ***: *P*<0.001.

A cell-binding study was performed to evaluate the Anxa1 targeting capacity of TMR-RIF7 in cells with high Anxa1 expression. Qualitative fluorescent imaging data show that cells treated with TMR-RIF7 (Fig. 4B) had greater fluorescent intensity than those treated with TMR-IF7 (Fig. 4A). In contrast, control peptide TMR-RQ7 (Fig. 4C) signals remained at background levels. In the inhibition assay, incubation with normal rabbit IgG (4 µg each well) prior to peptide treatment did not influence binding capacity (Fig. 4. D, G), whereas fluorescent signals were reduced significantly when cells were treated with anti-Anxa1 antibody prior to peptide treatment (Fig. 4 E, H). TMR-RIF7 (Fig. 4I) was more resistant to protease in complete medium than TMR-IF7 (Fig. 4F), which confirms data from the stability assay. In direct competition studies, compared with untreated TMR-IF7 (Fig. 5A) and TMR-RIF7 (Fig. 5B) groups, the fluorescent signal was significantly reduced when cells were treated with non-labeled peptides prior to treatment with TMR-labeled peptides (Fig. 5 C, D).

**Figure 6:**
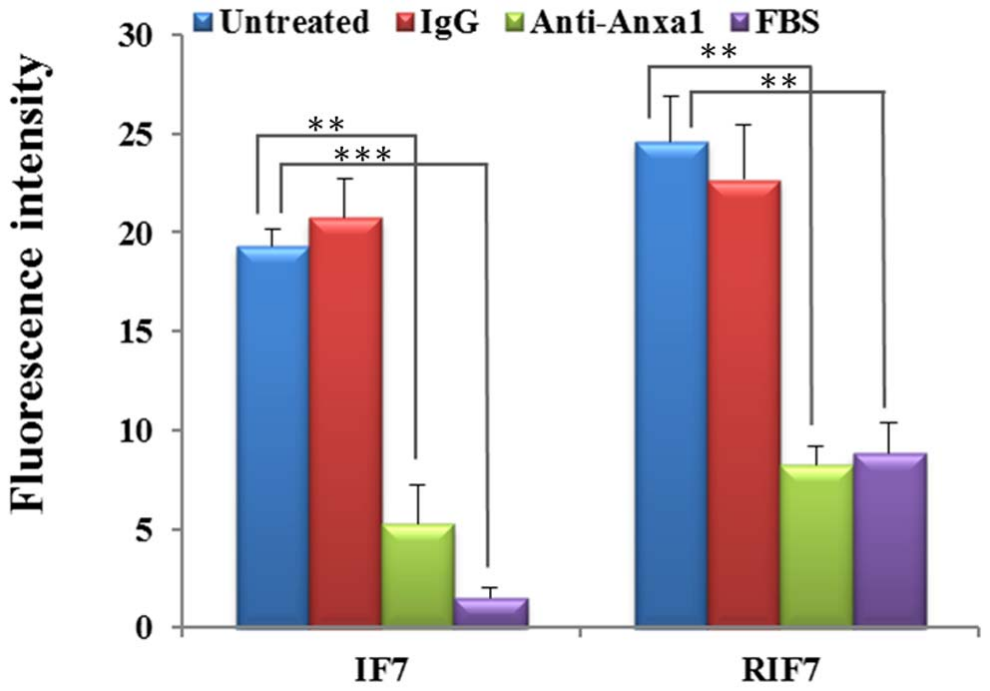
Fluorescent images of A549 cell for competition studies were observed with fluorescent microscopy. Cells were incubated for 30-IF7 (A) or TMR-RIF7 (B). Cells were incubated with non-labeled IF7 (C) or RIF7 (D) for 30 min in advance of TMR-labeled peptides. Quantitative cell-binding was measured using a F-7000 FL spectrophotometer. Data are presented as means ±SD (n = 3). **: *P*<0.01. Original magnification: ×20. Bar represents 100 µm.

The quantitative inhibition assay and the influence of FBS in the culture medium are depicted in Fig. 6. A significant difference (p<0.01) was observed between cells treated with only IF7 and those incubated with anti-Anxa1 antibody prior to IF7 treatment; the intensity of fluorescence decreased from 19.32±0.89 to 5.26±1.97. Similar results were found in the RIF7 group; ∼70% of the binding to Anxa1 was inhibited. In contrast, normal rabbit IgG had little influence on the binding capacity of IF7 or RIF7 to cells. When IF7 or RIF7 was incubated with complete medium (containing 10% FBS) for 10 min prior to incubation with cells, the fluorescent intensity decreased significantly (p<0.001) in the IF7 group and in the RIF7 group (p<0.01) IF7 is more susceptible to proteolysis in FBS than RIF7, and this was confirmed with the stability assay. Quantitative data from the competition assay is depicted in Fig. 5E which shows a significant difference (p<0.01) between cells treated only with IF7 and incubated with non-labeled antibody prior to IF7: the fluorescent intensity decreased from 20.31±1.78 to 4.26±1.96. Similar results were observed in the RIF7 group; ∼67% of the binding to Anxa1 was inhibited. These data agree with results of the inhibition assay, suggesting that retro-inverso peptide RIF7 has an equivalent receptor binding site as the parent peptide IF7.

### In vivo tumor-targeting activity by TMR-labeled peptides

**Figure 7:**
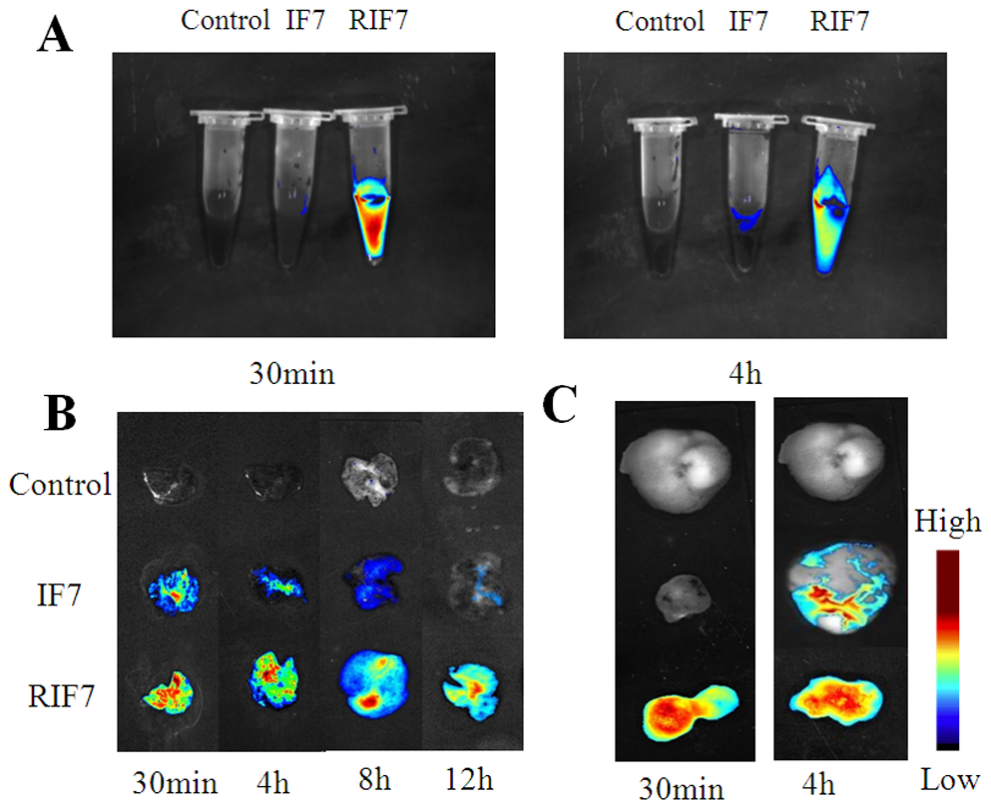
Fluorescent images of mouse blood and *ex vivo* tumor tissues. Fluorescent images of mouse blood bearing pulmonary metastasis at 30-IF7 or TMR-RIF7 (A). Representative *ex vivo* images of lung with melanoma at 30 min, and 4, 8, and 12 h (B). Representative *ex vivo* images of subcutaneous KBv xenografts at 30 min and 4 h after intravenous injection of TMR-IF7 or TMR-RIF7 (C).

**Figure 8:**
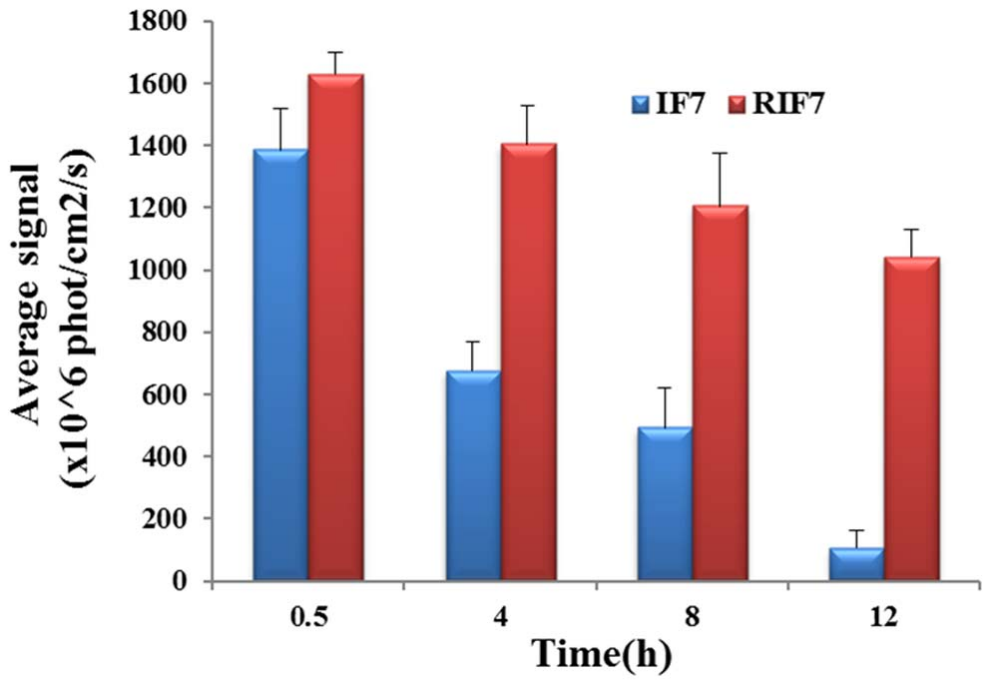
Half-quantitation of fluorescent intensity in lung melanoma as a function of time. Data are represented mean±SD (n = 3).

The fate of TMR-labeled peptides *in vivo* was determined in a metastatic pulmonary melanoma model and a subcutaneous KBv xenograft model using real-time NIR imaging systems. As shown in Fig. 7A, IF7 peptide was virtually degraded in mouse blood in 30 min after an intravenous injection whereas RIF7 was observed in the circulation after 4 h. These data show that IF7 was unstable *in vivo* compared with RIF7 and this might be attributing to the high susceptibility of IF7 to protease in the plasma. This also suggests that a retro-inverso modification can significantly increase its stability, a hypothesis that is confirmed with the stability assay *in vitro* data. Fig. 7B depicts the amount of IF7 and RIF7 in the lung melanoma and this amount is time dependent: 30 min post-injection, a maximum fluorescent signal was exhibited and this signal decreased over time. The RIF7 treatment group had a stronger fluorescent signal at all survey points compared with the IF7 group. Consistent results were observed in the subcutaneous KBv xenograft model (Fig. 7C). A greater fluorescent intensity was observed in tumors of RIF7-treated mice compared to IF7-treated mice at both 30 min and 4 h. The data may be explained by the fact that RIF7 was more stable than IF7 and consequently circulate longer and accumulate to a greater degree in tumor tissue. The mean fluorescent intensities in the lung tumor region over time are depicted in Fig. 8. Average signals of both IF7 and RIF7 in tumor tissues decreasing with time; meanwhile, the IF7 signal decreased more rapidly than RIF7. After 4 h, the signal intensity of RIF7 was ∼2.3-fold than that of IF7. Moreover, TMR-labeled RIF7 remained high (1042.89±86.02) whereas IF7 was hardly detectable at 12 h post intravenous injection. Therefore, possibly, the retro-inverso configurations have greater tumor targeting ability than the parent peptide IF7. Perhaps RIF7 has a prolonged circulation time due to its greater resistance to proteolytic enzymes *in vivo*.

**Figure 9:**
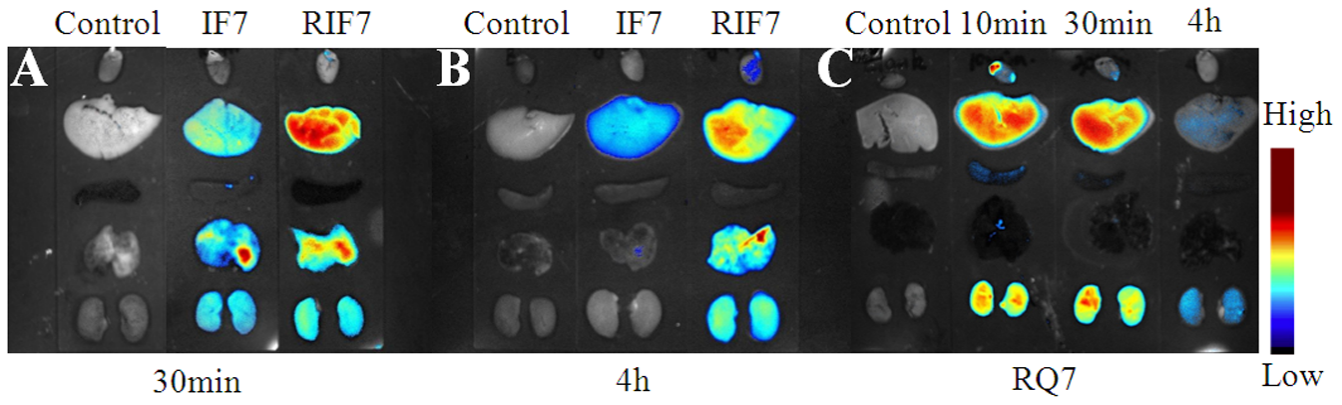
Fluorescent images of *ex vivo* organs. Representative ex vivo images of dissected organs (from top to bottom: heart; liver; spleen; lung; kidney) at 30 min after intravenous injection of TMR-IF7 or TMR-RIF7 (A). Representative ex vivo images of dissected organs (from top to bottom: heart; liver; spleen; lung; kidney) at 4 h after intravenous injection of TMR-IF7 or TMR-RIF7 (B). Representative ex vivo images of dissected organs (from top to bottom: heart; liver; spleen; lung; kidney at different time points after intravenous injection of TMR-RQ7 (C).

To evaluate the systemic distribution of TMR-labeled peptide *in vivo*, the major organs were collected at 30 min and 4 h after an intravenous injection of IF7 or RIF7. Both peptides were distributed mainly in liver, kidney and tumor tissue, and RIF7-treated animals had stronger fluorescent signals at both time points (Fig. 9 A, B). The behavior of RQ7 *in vivo* indicated tumor target distribution, rather than normal tissue distribution was responsible for the accumulation of IF7 or RIF7 in the lung; RQ7 signals in the lung remained at background levels (Fig. 9C).

**Figure 10:**
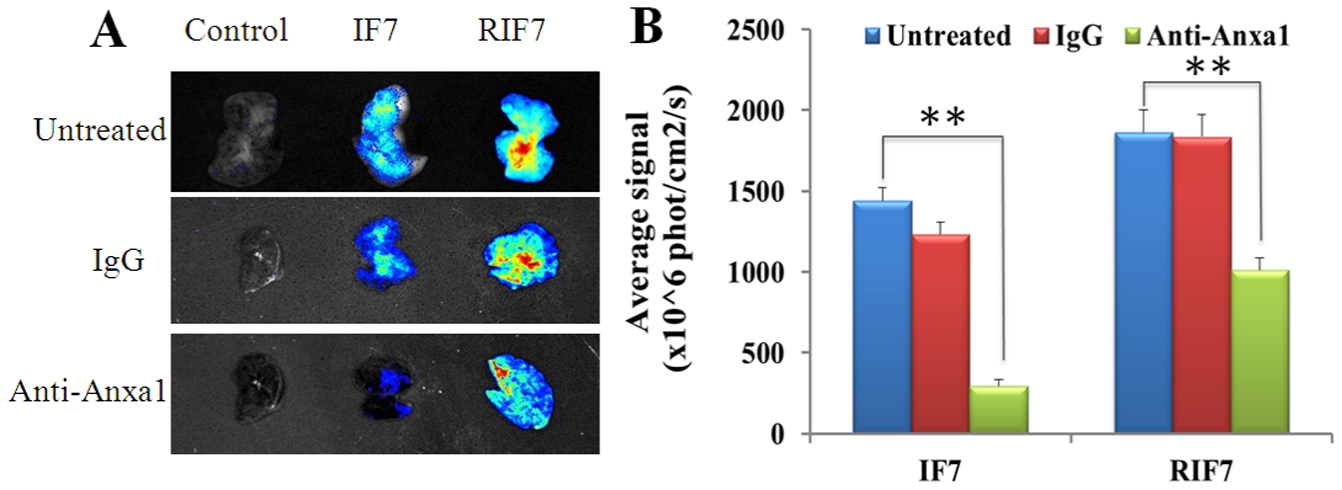
*Ex vivo* images of lung melanoma at 10 min for inhibition assay (A). Half-quantitation of fluorescence for inhibition assay (B). Data are represented mean±SD(n = 3). **: *P*<0.01.

For the inhibition assay, the injection of normal rabbit IgG prior to IF7or RIF7 did not alter targeted distribution in the lung. In contrast, fluorescent signals decreased significantly (p<0.01) with anti-Anxa1 antibody treatment (Fig. 10 A). As shown in Fig. 10B, the mean fluorescent intensities in the tumor region were reduced 79.70% and 45.62% for IF7 and RIF7, respectively, when treated with anti-Anxa1 antibody prior to TMR-labeled peptides, strongly suggesting that IF7 or RIF7 targets tumors through Anxa1 expressed on endothelial cell surfaces. Also, the binding ability for Anxa1 was more potent for RIF7 than for IF7, so retro-inverso modification of IF7 improved its targeting ability with its corresponding receptor—Anxa1 unchanged.

Retro-inverso peptides have been reported to imitate the bioactivity of natural L-peptides, such as those in immunological responses [23], [24] or serve as valuable probes for detecting antibodies [25]. These observations may be explained by the similar topological structure of retro-inverso peptides and their parent peptides; they are mirror images of one another [26]. An alternative hypothesis is that retro-inverso modification returns side chains to their original positions in space thus approximately maintaining the topology of them. Side chains are likely more important for defining the antigenic specificity than the main chain [27]. Guichard and co-workers suggested that retro-inverso antigenic mimicry occurs only when retro-inverso peptides are in random coil, loop, or cyclic conformations [28]. So it is reasonable to speculate the structure of RIF7 is one of these three. Although the mechanisms of antigenic mimicry between retro-inverso peptides and parent peptides still remain unclear, our modification methods pave a novel path for the future development of therapeutic applications of peptides.

With respect to the targeted therapeutics presently developed, most research has concentrated on directly killing individual tumor cells; thus, internal solid tumor cells were not as accessible to anti-cancer drugs. Another obstacle for targeted therapeutics is the intrinsic genetic instability of cancer cells, which produce heterogeneous patterns of tumor marker expression [29]. Targeted drug delivery to the tumor endothelium is a more practical strategy for inhibiting tumor progression. Abundant receptors are more accessible and stable. Therefore, identification of markers up-regulated within the tumor endothelium, together with the generation of corresponding binding moieties, offers a promising novel therapeutic strategy to target specific moieties to the tumor environment.

Previous research indicates that carbohydrate mimetic peptide IF7 has a remarkable targeting capacity forAnxa1, which is a specific marker expressed on the tumor endothelium. Because peptide-based therapeutics are susceptible to proteases, which occurred with IF7 [30], [31], retro-inverso modification is a commonly used method to increase peptide stability. Compared with IF7, RIF7 not only accumulated more in tumor tissues but also accumulated in normal tissues including the liver and kidney. This was likely largely due to improved stability. However, accumulation in the liver decreased more than in lung tumors at 4 h, suggesting that improved stability of RIF7 would contribute more to tumor distribution of the peptide compared to normal tissues. Thus, RIF7 should function better for targeted drug delivery for tumor therapy.

## Conclusion

In our study, retro-inverso modification was employed to confer protease resistance to IF7 while retaining targeting capacity. The modified peptide RIF7 has significantly increased stability and identical binding receptor compared with the parent peptide IF7. Thus, RIF7 deserves further research into its capacity as a drug delivery vehicle that targets Anxa1 which is over-expressed on the surface of the tumor vasculature.
